# Rate-constraining changes in surface properties, porosity and hydrolysis kinetics of lignocellulose in the course of enzymatic saccharification

**DOI:** 10.1186/s13068-016-0431-3

**Published:** 2016-01-26

**Authors:** Ville Pihlajaniemi, Mika Henrikki Sipponen, Anne Kallioinen, Antti Nyyssölä, Simo Laakso

**Affiliations:** Aalto University, School of Chemical Technology, P.O. Box 16100, FI-00076 Espoo, Finland

**Keywords:** Enzymatic lignocellulose hydrolysis, Cellulose surface, Lignin surface, Pore size distribution, Cellulases, Kinetic modelling, Inhibition

## Abstract

**Background:**

Explaining the reduction of hydrolysis rate during lignocellulose hydrolysis is a challenge for the understanding and modelling of the process. This article reports the changes of cellulose and lignin surface areas, porosity and the residual cellulase activity during the hydrolysis of autohydrolysed wheat straw and delignified wheat straw. The potential rate-constraining mechanisms are assessed with a simplified kinetic model and compared to the observed effects, residual cellulase activity and product inhibition.

**Results:**

The reaction rate depended exclusively on the degree of hydrolysis, while enzyme denaturation or time-dependent changes in substrate hydrolysability were absent. Cellulose surface area decreased linearly with hydrolysis, in correlation with total cellulose content. Lignin surface area was initially decreased by the dissolution of phenolics and then remained unchanged. The dissolved phenolics did not contribute to product inhibition. The porosity of delignified straw was decreased during hydrolysis, but no difference in porosity was detected during the hydrolysis of autohydrolysed straw.

**Conclusions:**

Although a hydrolysis-dependent increase of non-productive binding capacity of lignin was not apparent, the dependence of hydrolysis maxima on the enzyme dosage was best explained by partial irreversible product inhibition. Cellulose surface area correlated with the total cellulose content, which is thus an appropriate approximation of the substrate concentration for kinetic modelling. Kinetic models of cellulose hydrolysis should be simplified enough to include reversible and irreversible product inhibition and reduction of hydrolysability, as well as their possible non-linear relations to hydrolysis degree, without overparameterization of particular factors.

**Electronic supplementary material:**

The online version of this article (doi:10.1186/s13068-016-0431-3) contains supplementary material, which is available to authorised users.

## Background

Hydrolysis of lignocellulose materials by cellulases is conceived as a complex, heterogenous multi-enzyme process with several inhibitory mechanisms, changing substrate properties and the kinetics generally obscured by several non-linearities. This is a challenge for fundamental understanding, as well as for process design in the utilisation of renewable plant biomass such as straw, bagasse, corn stover and wood residues for the production of biofuels and chemicals. Cellobiohydrolases bind to cellulose surface through a cellulose-binding module and catalyse hydrolysis of cellulose in a processive manner, moving along the cellulose surface and catalysing multiple hydrolysis reactions during a single run [1]. Hydrolysis is constrained by different inhibitory effects and the maximum degree of hydrolysis appears to depend on the enzyme dosage, suggesting that the enzyme activity is partly lost [2, 3]. While this is particularly visible with lignin-containing substrates, it also occurs with pure cellulose [4, 5]. There are essentially three kinetically different categories of effects slowing down the hydrolysis rate: permanent decrease of enzyme activity in the reaction either by irreversible non-productive binding of enzymes [1, 6, 7] or by denaturation [2, 8], reversible non-productive binding or product inhibition by sugars and other inhibitory products released during hydrolysis [9–11], and decrease in the hydrolysability of the substrate [1, 2, 7–12].

A major reason for the removal of enzyme activity from the reaction is the binding of cellulases onto lignin. However, the activity of the enzyme molecule is not lost [13], so strictly speaking this is not inhibition in the conventional sense. Binding of the cellulases to lignin and the interactions have been shown to be strong, possibly irreversible [6, 7, 14]. Non-productive binding, on the other hand, is not restricted to lignin [1]. Adsorption on cellulose is generally reversible and fast [15], but it does not always lead to hydrolysis and irregular cellulose structures may lead to unfavourable binding interactions, which may increase in proportion during hydrolysis. Although adsorption on lignin has been suggested to occur gradually [7], adsorption on actual lignocellulose substrates is fast compared to the common reaction times of several days, reaching equilibrium in less than an hour [16] and thereafter depending on the degree of hydrolysis [4, 17].

Thermal or mechanical denaturation is another reason for the loss of active enzymes during reaction. Denaturation has been observed at typical reaction temperatures for enzyme solutions in the absence of substrates, or due to shear forces at high mixing rates [2, 8, 18]. However, other studies observed no particular thermal denaturation during actual hydrolysis under the reaction conditions commonly used [5, 13].

Cellulases are susceptible to competitive product inhibition by glucose and particularly by cellobiose and xylooligomers [9–11]. Product inhibition may not be restricted to sugars, since other inhibitory compounds, such as phenols [11, 19–21] or lignin-carbohydrate complexes [14] may be released during hydrolysis.

The hydrolysability of cellulose decreases during hydrolysis, regardless of the presence of lignin [3, 4, 22]. In the classical view, the easily hydrolysable substrate is hydrolysed fast, leaving behind the more recalcitrant parts of cellulose. Previously this has been attributed to the fast hydrolysis of amorphous cellulose and accumulation of crystallinity [23, 24], but other accumulating hindrances assumably contribute. These include obstructions in the reaction path by irregularities in cellulose structure, steric hindrances by lignin and reducing cellulose accessibility [1, 12, 25, 26].

A majority of studies have focused on correlating hydrolysability to the initial material properties [25, 27–30], sometimes including comparison to the properties after hydrolysis [31]. While the changes that take place during hydrolysis are expected to affect kinetics [32], only few reports on the material changes as a function of reaction time or hydrolysis degree exist [33] and so far the development of the surface areas of lignin or cellulose, or porosity have not been described. While the hydrolysis degree depends on hydrolysis time, it is unclear whether the hydrolysis kinetics only depend on hydrolysis degree, or if there are also time-dependent changes in the substrate other than hydrolysis itself. Since lignin is amorphous and subject to phase transitions at high temperatures [7, 34], it could be hypothesised that coalescence or spreading of lignin may slowly occur also under hydrolysis conditions, affecting the available surface areas of lignin or cellulose.

Many kinetic models have attempted to describe the complexity and the different dynamic factors of lignocellulose hydrolysis [22–24, 32, 35–37]. These often result in high numbers of parameters, while the model may still deviate from experimental data. In a basic kinetic approach, Langmuirian adsorption of cellulases has been combined with different inhibitory factors [36], which is also the basis of the NREL model by Kadam et al. [24] that has received particular attention. The model includes inhibition by sugars, linear decrease in hydrolysability and separately models the behaviour of exo- and endocellulases, cellobiases and xylanases. However, the attempts to calibrate the model have shown overparameterization, with high uncertainty in the parameter meaningfulness [38]. In other kinetic approaches, simplifications have been introduced for developing mechanistic models with decent fit and a low number of parameters [37]. However, instead of producing models as the primary aim, the robust mechanistic models could also be used for studying the outlines of the major dynamic effects during hydrolysis.

This article reports the change in the surface areas of lignin and cellulose, as well as in the pore size distribution of autohydrolysed or delignified wheat straw, phenol dissolution during hydrolysis and the role of the phenolics in product inhibition. The effect on the different factors on hydrolysis kinetics and residual enzyme activity is studied and the time dependence of the changes is considered. Taking a step back from the complexity of lignocellulose hydrolysis, a simplified and robust kinetic modelling approach is applied for the comparison of different potential rate-constraining factors.

## Results and discussion

### Hydrolysis reactions

**Fig. 1:**
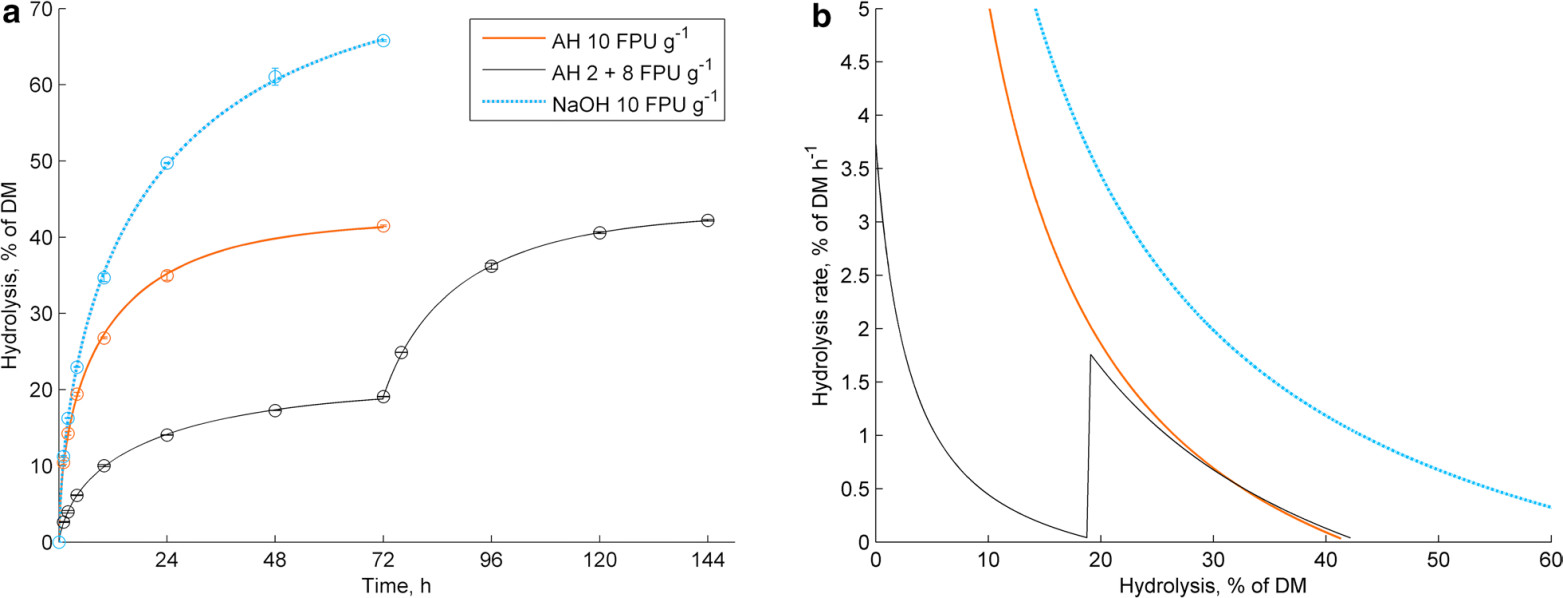
Hydrolysis time curves (**a**) and hydroysis rates as the function of hydrolysis degree (**b**) for 10 FPU g^−1^ enzyme dosage with NaOH-straw (*blue-dotted line*), AH-straw (*red solid line*) and the stepwise reaction with AH-straw with initial 2 FPU g^−1^ for 72 h, a subsequent addition of 8 FPU g^−1^ and continued incubation for another 72 h (*black line*). *Error bars* represent the standard deviation of duplicate hydrolysis reactions

Enzymatic hydrolysis was performed to autohydrolysed straw (AH-straw, 51.7 % glucan, 3.4 % xylan, 30 % Klason lignin) and NaOH-delignified straw (NaOH-straw, 78.9 % glucan, 10.3 % xylan, 3.8 % Klason lignin) with an enzyme dosage of 10 FPU g^−1^ (per dry matter, DM) for 72 h. The hydrolysis reactions showed typical asymptotic time curves of cellulose hydrolysis, (Fig. 1a) leading to the hydrolysis of 65 % of the NaOH-straw DM and 45 % of the AH-straw DM. For studying the possible time-dependent effects on the substrate, AH-straw was hydrolysed with stepwise enzyme addition with the rationale that a slow hydrolysis prior to a second enzyme addition would allow possible time-dependent events to take place in the substrate, compared to the fast reaction with the direct addition of 10 FPU g^−1^ enzyme dosage. First the AH-straw was hydrolysed with 2 FPU g^−1^ for 72 h, reaching almost half of the degree of hydrolysis obtained with 10 FPU g^−1^, which reflected the non-linear correlation of hydrolysis degree to cellulase dosage [2, 3]. Subsequently, the remaining 8 FPU g^−1^ was added and the hydrolysis was continued for another 72 h. The final hydrolysis degree turned out to be equal to that of the direct hydrolysis by 10 FPU g^−1^. This suggest that the hydrolysis rate depends exclusively on the hydrolysis degree and no other time-dependent effects on the substrate affect the hydrolysability than hydrolysis itself. Therefore, each portion of enzymes provides its contribution and faces inhibitory effects independently, regardless of the history of the hydrolysis. This is further corroborated by studying the hydrolysis rate as a function of hydrolysis degree (Fig. 1b), which is almost equal for the direct reaction with 10 FPU g^−1^ and for the stepwise reaction after the second enzyme addition. The initial hydrolysis rate with 2 FPU g^−1^ is higher than the rate immediately after the 8 FPU g^−1^ addition at the 20 % hydrolysis degree, indicating accumulation of hydrolysis-dependent inhibitory effects and decreasing hydrolysability, rather than time-dependent denaturation or gradual non-productive binding. This is in accordance with the previously reported low denaturation under actual reaction conditions [5, 13], although gradual enzyme adsorption on pure lignin preparations has been reported [7].

For the sake of comparability, it should be noted that a portion of 2 FPU g^−1^ was incubated for an extra 72 h, so a small increase in hydrolysis could have been expected. However, extending the reaction time generally has a small effect on cellulose hydrolysis [2, 4, 5], and further so, if it only concerns the portion of 2 FPU g^−1^.

### Changes in cellulose and lignin surfaces and dissolution of phenols

**Fig. 2:**
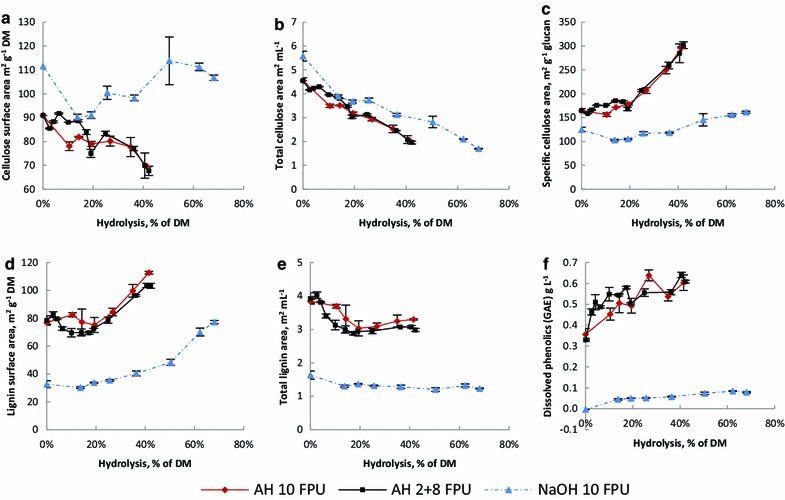
Surface areas of cellulose and lignin and dissolution of phenols as a function of hydrolysis. **a** Cellulose area per DM, **b** the total cellulose area per mL in the reaction, **c** specific cellulose surface area, **d** lignin area per DM, **e** total lignin area per mL reaction, **f** dissolved phenols (gallic acid equivalent, GAE). *Error bars* represent the standard deviation of the analysis of duplicate reactions

The surface areas of cellulose and lignin (accessible phenolic hydroxyls) were determined in the course of hydrolysis by determining the adsorption maxima of the dyes Congo Red and Azure B on the material, respectively [25, 39]. The cellulose area per DM of AH-straw was decreased from 90 to 68 m^2^ g^−1^ and the cellulose area of NaOH-straw (Fig. 2a) first rapidly decreased from 112 to 90 m^2^ g^−1^, possibly representing removal of amorphous cellulose or collapse of the material structure, and then eventually increased close to the initial value. While the surface area per DM describes the changes in the material, it is more relevant for hydrolysis kinetics to describe the total area available in the reaction suspension (m^2^ per mL). The total cellulose area per mL was most affected by mass reduction of the substrate by hydrolysis, decreasing from 4.6–5.6 to 1.7–2.0 m^2^ per mL with both substrates (Fig. 2b). For enzyme kinetics, the cellulose area has been considered to represent the substrate concentration better than the total carbohydrate content in the material [24, 32, 36]. However, since the cellulose area per mL shows a roughly linear correlation with hydrolysis degree, the carbohydrate content seems to be an appropriate approximation of the substrate concentration after all. The specific cellulose area (m^2^ per g cellulose) indicates changes in the cellulose shape and association with other lignocellulose components. The specific cellulose area was increased by hydrolysis, particularly with AH-straw (Fig. 2c), where an increase from 165 to 302 m^2^ per g cellulose was observed. This may reflect in increasing cellulose surface roughness and thinning of cellulose crystals by hydrolysis occurring on a particular side [40], which may be emphasised in crystals partially embedded in lignin. It has been suggested that only 2 % of total cellulose is located at accessible fibril surfaces [32]. Hydrolysing a cellulose molecule on the crystal surface reveals fresh surface underneath and the total area thus depends on the shape and roughness of the crystals and the proportion of sterically hindered cellulose. In accordance with these results, surface roughness of cellulose has been reported to increase during hydrolysis [33, 40].

The lignin surface area of the substrates was increased by hydrolysis due to the increase in lignin proportion (Fig. 2d). It would seem plausible that hydrolysis of cellulose would reveal fresh lignin surface, which would lead to increased non-productive binding as a function of hydrolysis degree. However, it turned out that the total surface area of lignin in the reaction was not increased during hydrolysis, but was in fact decreased in the early phase of the reaction (Fig. 2e). The decrease for AH-straw was from 4.0 to 2.9 m^2^ per mL and for NaOH-straw from 1.6 to 1.2 m^2^ per mL, after which the areas remained relatively unchanged. The decrease was explained by the observation of simultaneous dissolution of phenolics (Fig. 2f). If non-productive binding of cellulases on lignin is increased with increasing hydrolysis degree, it appears to be rather a consequence of increased accessibility to lignin than change in the actual lignin area. Another explanation could be the previously suggested gradual binding of enzymes on lignin [7], or the combination of both. However, the changes in the amount of residual soluble activity in the hydrolysates do not support substantial time dependence of activity loss, as explained below.

The only event that was observed to be partly time dependent was the dissolution of phenolics. A higher extent of dissolution of phenolic substances is observed during the early stages of the slow 2 FPU g^−1^ reaction of AH-straw compared to the faster 10 FPU g^−1^ reaction (Fig. 2e). As it took a longer time for the 2 FPU g^−1^ reaction to reach the equal hydrolysis degree as the 10 FPU g^−1^ reaction, time seems to be the apparent factor behind the higher dissolution of phenolics. This suggests that it may not have been a direct result of hydrolysis, but rather extraction facilitated by hydrolysis. The higher phenol dissolution in the 2 FPU g^−1^ reaction coincides with a larger decrease in lignin surface and smaller decrease in cellulose surface, indicating that a small cellulose surface area was uncovered by the dissolution of phenolics. However, these differences in the total areas of cellulose and lignin per mL are too small to cause observable differences in hydrolysis. Eventually, the surface areas of cellulose and lignin, as well as phenol dissolution reach equal values for both the slow and fast hydrolysis of AH-straw. This further affirms the conclusion that hydrolysis is not affected by time-dependent changes in the substrate, thus ruling out the hypothesis of coalescence or spreading of lignin affecting cellulose accessibility.

### Residual cellulase activity and product inhibition

**Fig. 3:**
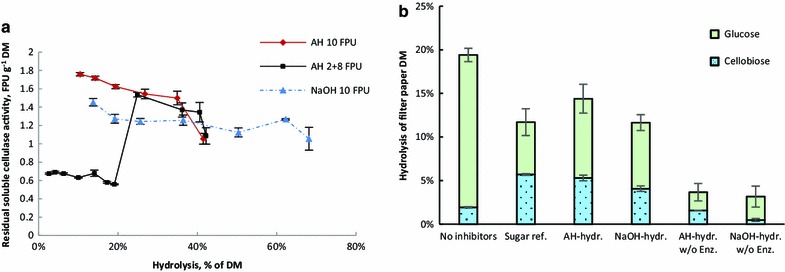
**a** Residual soluble cellulase activity from the original 10 FPU g^−1^ as a function of hydrolysis degree. **b** Product inhibition in the presence of hydrolysates or pure sugars

The residual soluble enzyme activity was studied for implications of irreversible or reversible activity loss. Filter paper was hydrolysed with the hydrolysate supernatant samples and the resulting hydrolysis degree was converted into a corresponding enzyme amount by a non-linear hydrolysis standard produced with fresh enzymes. Product inhibition by the hydrolysate sugars is not expected to affect the observed residual activity, since the hydrolysate samples were diluted tenfold for the analysis reaction (maximally 2.4 and 3.9 g L^−1^ total sugars in AH- and NaOH-straw hydrolysates, respectively). The majority of cellulases was adsorbed to the substrates after 1 h of hydrolysis, in accordance with previous reports [4, 17]. Of the initial 10, 1.8 and 1.5 FPU g^−1^ soluble activities were observed after 1 h of hydrolysis with AH- and NaOH-straw, respectively, and of the 2 FPU g^−1^ initial dosage, 0.67 FPU g^−1^ remained soluble (Fig. 3a). The residual activity decreased during hydrolysis and with AH-straw the correlation was roughly linear with hydrolysis degree. With AH-straw the rate of activity loss was higher compared to NaOH-straw, and the residual activity in all reactions ended up equal, 1.1 FPU g^−1^. Immediately after the second enzyme addition in the stepwise reaction, similar residual activities were observed as with the direct initial 10 FPU g^−1^ dosage, suggesting that no significant denaturation had occurred during the 2 FPU g^−1^ hydrolysis.

The residual activity appears to decrease linearly with increasing hydrolysis degree, supporting the assumption of hydrolysis-dependent inhibitory mechanism and suggesting a dynamic equilibrium, i.e. reversible inhibition by the solid fraction. Considering the possibility of irreversible binding on lignin emerging as a function of hydrolysis, the available lignin should bind enzymes to full capacity, until enzymes are depleted. Irreversible binding should therefore cause a higher proportional activity loss rate for the smaller enzyme dosage, compared to the higher. This is not supported by the results, which show similar proportional rates of activity loss for both enzyme dosages. On the other hand, if only the initial lignin surface is considered and binding is assumed significantly time dependent [7], the residual activity should follow exponential decay similar to denaturation, which is also not observed. However, the decreasing substrate amount should lead to simultaneous release of enzymes and the actual activity loss may therefore be higher and less linear than observed. Therefore the possibility of time-dependent activity loss cannot be completely ruled out and could be explained by the combinatory effect of hydrolysis-dependent increase in accessible lignin surface and time-dependent binding. However, previous reports [4, 17] have shown somewhat stable residual activities after the initial rapid changes. It seems unlikely that this would be the result of non-linear effects of enzyme release by substrate consumption and time-dependent activity loss cancelling each other out.

Part of the activity loss may also be the result of increasing amount of non-productive binding sites on cellulose, such as cellulose partially embedded in lignin or other irregular structures where hydrolysis cannot proceed. While such occurrences have been observed [1], no method for their quantification yet exists.

Dissolved phenolic substances have been shown to inhibit cellulases [11, 21], and the observed dissolution of phenolics could contribute to “product inhibition” as well as sugars. The presence of soluble non-sugar inhibitors was therefore studied by hydrolysis of filter paper in the presence of the hydrolysates from the 72 h (10 FPU g^−1^) reactions with an addition of fresh enzymes at 10 FPU g^−1^. The hydrolysis was compared to reference reactions with or without an equal concentration of pure sugars. The initial concentrations of glucose and xylose were equalised to those of the reaction with the NaOH hydrolysate by the addition of pure sugars (29.4 g L^−1^ glucose and 2.5 g L^−1^ xylose). In reference reactions without the hydrolysates, this sugar concentration showed a typical product inhibition pattern, with increased cellobiose concentration indicating inhibition of β-glucosidases and an overall yield decrease of 40 % indicating β-glucanase inhibition (Fig. 3b) [24, 41]. Compared to the sugar reference, a slightly higher hydrolysis yield was achieved with the AH hydrolysate and an equal hydrolysis yield with the NaOH hydrolysate. While the higher yield is attributed to the residual activity in the hydrolysates, it also suggests that the phenols found in the AH hydrolysate did not substantially contribute to inhibition. Surprisingly, the NaOH-straw hydrolysate seemed slightly more inhibitory than the AH-straw hydrolysate, showing a lower hydrolysis yield compared to the AH-straw hydrolysate. However, this may also be explained by differences in the residual enzyme proportions. It could be hypothesised that the higher cellulose content in delignified straw may have led to a higher proportion of non-productive binding of exoglucanases on irregular cellulose structures. However, further elucidation for this issue is required. In the reactions without fresh enzyme addition, the differences in cellobiose concentrations suggested that the NaOH-straw hydrolysate contained a higher proportion of β-glucosidases compared to the AH-straw hydrolysate. This is consistent with the lower affinity of β-glucanases on cellulose and less-specific binding of lignin [7].

### Effect of hydrolysis on pore size distribution

**Fig. 4:**
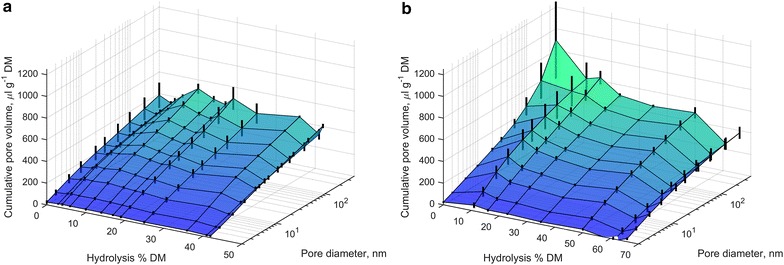
Effect of hydrolysis on pore size distributions of AH-straw (**a**) and NaOH-straw (**b**)

Substrate accessibility has been considered to be an important factor in lignocellulose hydrolysis [25, 26]. Therefore, the changes in pore size distribution during hydrolysis were studied by thermoporometry. Initially, NaOH-straw showed a considerably higher overall porosity compared to AH-straw, which is in accordance with our recent work [42], where the porosity was found to correlate with hemicellulose content of delignified straw. In the course of hydrolysis, a decreasing trend in porosity was observed for NaOH-straw (Fig. 4). For AH-straw, however, no detectable trend was observed. It may be that hemicellulose hydrolysis leads to the collapse of the pores in NaOH-straw, while a base level of porosity is maintained by lignin in AH-straw. In any case, increasing accessibility of enzymes to lignin in AH-straw cannot be concluded from these results.

### Kinetic modelling of hydrolysis and inhibition

Instead of an all-inclusive hydrolysis model, this work was aimed at assessing the compatibility of the observed hydrolysis with different mechanisms that may constrain lignocellulose hydrolysis during the course of the reaction. The model follows the idea of cellulose hydrolysis consisting of adsorption of cellulases, and subsequent hydrolysis [24, 36]. Since adsorption and processive catalysis take place independently, E does not appear on the product side of the reaction equation (Eq. 1), as it would with Michaelis–Menten kinetics. The adsorption is assumed to follow Langmuir kinetics (Eq. 2, [24]) and the catalysis rate is assumed to be first order, with the catalytic constant *k*_cat_ (Eq. 3). The *k*_cat_ describes the average rate of catalysis by adsorbed cellulases and is assumed to be substrate dependent. Instead of elaborating on a multi-enzyme reaction, the overall catalysis by exo- and endoglucanases is considered rate limiting and modelled as a single reaction, while β-glucosidase and xylanase activities are considered accessory and adequate.

1 $$E + S {\mathop{\leftrightarrow}\limits_{k_{-1}}^{k_{1}}} {\text{ES}} {\mathop{\rightarrow}\limits_{}^{k_{\text{cat}}} }P$$

2 $$[{\text{ES}}] = \frac{{\left[ S \right]e_{m} [E_{\text{F}} ]K}}{{1 + [E_{\text{F}} ]K}}$$

3 $$r = k_{\text{cat}} \left[ {\text{ES}} \right]$$

After the formation of the substrate–enzyme complex [ES], it is assumed that the rate is no more dependent on the substrate concentration and thus [*S*] is not included in the reaction equation, diverging from the NREL model. As this would lead to second-order reaction rate i.e. exponential rate decrease with decreasing substrate concentration, the sources of the rate decrease may be better discriminated by assuming first-order catalysis rate with the addition of rate-constraining effects separately. Substituting the free enzyme concentration [*E*_F_] = [*E*_0_]−[ES] to the adsorption equation and solving [ES] from the rate equation leads to a quadratic equation, which has two positive roots at positive values of the adsorption constant K and the amount of adsorption sites per substrate, *e*_*m*_, of which the smaller is realistic, while the larger is impossible ([ES] > [*E*_0_]). This model (Eq. 4) can be fitted to hydrolysis results without separately determining the adsorption parameters.

4 $$[{\text{ES}}] = \frac{{-b + \sqrt {b^{2} - 4ac} }}{2a}$$

$$a = -K; b = 1 + K[E_{0} ] + [S]e_{m} K; c = - [S]e_{m} K[E_{ 0} ]$$

Starting from this model, different rate-constraining mechanisms may now be incorporated separately or in combinations. The constraining factors, denoted by *I*, are defined separately for each mechanism and assumed to emerge as a linear function of hydrolysis degree (Eq. 5). In the case of inhibition, the inhibition constant (*K*_*I*_; [24]) is already accounted for by the empirical constant α and is thus excluded.

5 $$I = \frac{{\alpha \left( {\left[ {S_{0} } \right]-[S]} \right)}}{{[S_{0} ]}}$$

Reversible product inhibition is formulated similarly as in the NREL model, but instead of discriminating between different sugars, the different sources of inhibition are combined into a single factor, which accounts for sugars, other soluble inhibitors and non-productive binding sites on solids that may emerge during hydrolysis (Eq. 6). This simplification assumes equal affinity to all inhibitors.

6 $$r = \frac{{k_{\text{cat}} \left[ {\text{ES}} \right][S]}}{1 + I}$$

Irreversible inhibition means permanent removal of enzymes from the reaction, by the formation of a permanent enzyme–inhibitor complex [EI], for example by non-productive adsorption. Although it is normally considered time dependent, in the time frame of lignocellulose hydrolysis it can be simplified to occur instantaneously, as the inhibitor emerges ([EI] = *I*). Assuming that the concentration of the inhibitors increases linearly with hydrolysis, the inhibition can be described as a change in the total enzyme amount as a function of hydrolysis (Eq. 7). [*E*_0_] is thus defined as the total concentration of enzymes that have not been permanently inhibited. On the other hand, the possible time dependence of the formation of [EI] can be included as proportional to the free enzyme ([*E*_F_] = [*E*_0_] − [ES]) concentration and the concentration of free inhibitor binding sites (*I − *[EI]) with a rate constant *λ* (Eq. 8).

7 $$[E_{0} ] = \left[ {E_{{0,{\text{initial}}}} } \right] - [{\text{EI}}]$$

8 $$\frac{{{\text{d}}[{\text{EI}}]}}{{{\text{d}}t}} = \lambda \left( {\left[ {E_{0} } \right] - [{\text{ES}}]} \right)\left( {I - [{\text{EI}}]} \right)$$

Exoglucanases processively hydrolyse long stretches of cellulose molecules along available paths, halting at obstructions, which potentially stops the catalysis run [1]. Decrease in substrate hydrolysability may be described as an increase of such obstructions, and thus as a decreasing average rate of catalysis, *k*_cat_ (Eq. 9).

9 $$k_{\text{cat}} = k_{\text{cat,initial}} -I$$

Thermal (or mechanical) denaturation leads to time-dependent first-order reduction (exponential decay) in the total enzyme amount, regardless of the hydrolysis degree (Eq. 10), according to a rate constant λ.

10 $$\frac{{{\text{d}}E_{0} }}{{{\text{d}}t}} = - \lambda [E_{0} ]$$

### Comparison of different inhibition models

**Fig. 5:**
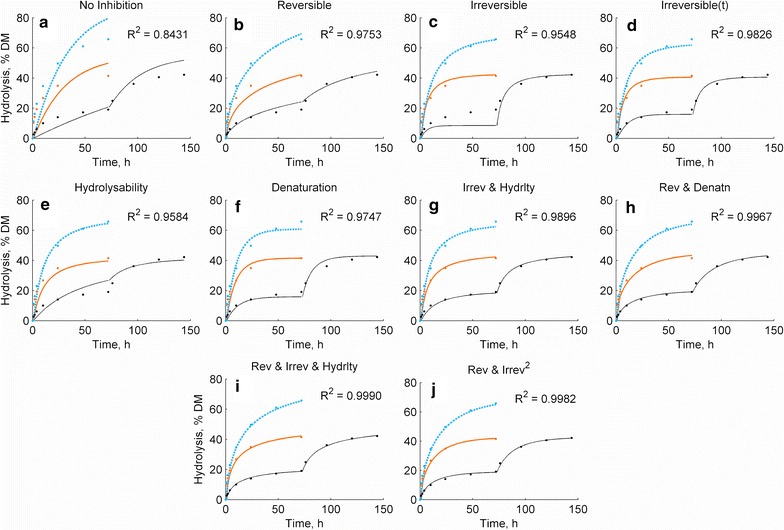
Hydrolysis model predictions (*lines*) and actual data points (*dots*). Colour meanings are the same as in Fig. 1. No inhibition (**a**), Separately incorporated constraining factors: reversible (**b**) or irreversible (**c**) product inhibition, time-dependent irreversible product inhibition (**d**), denaturation (**e**) and reduction of hydrolysability (**f**). Combinations of factors: Irreversible product inhibition and decrease of hydrolysability (**g**), reversible product inhibition and denaturation (**h**), the combination of reversible and irreversible product inhibition and reduction of hydrolysability (**i**), and linear reversible and exponential (quadratic) irreversible product inhibition (**j**). The standard deviations were the same order of magnitude as the size of the symbols used to mark the data points

Different kinetic models were iteratively fitted to the hydrolysis time curves. Fitting to time curves has been concluded to be more appropriate for cellulase kinetics than conventional initial rate determinations of lignocellulose hydrolysis, since the initial rates include no information about the changes in the substrate hydrolysability in the course of the reaction [24, 32]. The substrate concentration [S] was represented by the total amount of cellulose in the reaction, which is a decent approximation, as shown above. By studying the systematic divergence of a kinetic model from the observed hydrolysis, it was possible to illustrate the characteristics of alternative rate-constraining mechanisms. The basic hydrolysis model with four parameters included Langmuirian adsorption of the enzymes on cellulose, followed by first-order catalysis with a substrate-dependent catalytic constant, *k*_cat_. Fitting the model without inhibitory factors showed high systematic divergence from the actual results (*R*^2^ = 0.843; Fig. 5a), since reduction in the substrate concentration could not alone explain the reduction in the reaction rate. In order to compare the alternative inhibitory mechanisms, they were first incorporated to the model separately (five parameters, *R*^2^ from 0.955 to 0.975). Reversible product inhibition was expected to include the combined effect of linearly increasing amount of sugars, phenols and non-productive binding etc. However, the actual decrease of hydrolysis rate was too drastic to be explained by reversible product inhibition alone (Fig. 5b). In accordance with the known cellulase behaviour [2, 3], the 2 and 10 FPU g^−1^ reactions appear to approach different asymptotes, which is not expected from reversible enzyme inhibition. Besides, as presented above, the presence of a previous hydrolysate only led to a 40 % decrease in the hydrolysis of fresh substrate, which is far from complete inhibition. On the other hand, irreversible product inhibition, which could include irreversible binding on an increasing accessible lignin surface area, showed a steeper rate decrease than actually observed, and a complete depletion of the 2 FPU g^−1^ enzyme dosage early in the reaction (Fig. 5c). If the irreversible binding was allowed to be time dependent (Fig. 5d, six parameters), an increased fit was obtained (*R*^2^ = 0.983) but a similar systematic error remained. The effect of denaturation on the predicted hydrolysis rate was also too steep (Fig. 5e) and led to an almost complete loss of activity at all enzyme dosages. The final major potential effect, reduction of hydrolysability, showed similar effects as reversible inhibition, with underestimated rate decrease and inadequacy to describe the different asymptotes of different enzyme dosages (Fig. 5f).

An increased fit (*R*^2^ > 0.99, 6 parameters) with a low systematic divergence required a combination of permanent activity loss and a moderate hydrolysis-dependent constraint, such as irreversible product inhibition and reduction of hydrolysability (Fig. 5g), or denaturation and reversible product inhibition (Fig. 5h). It appears that the different asymptotes at different enzyme loadings may only be explained by partial permanent activity loss, even though it was not apparent from the residual activities presented above. The current and previous evidence [5, 7, 13] seems to favour irreversible inhibition rather than denaturation. It therefore seems plausible to include irreversible inhibition in lignocellulose hydrolysis models, in addition to the known effects of product inhibition and decreasing hydrolysability. The combination of all three factors led to a 7-parameter model with the best fit of this study (*R*^2^ = 0.9990; Fig. 5i). This leads to the conclusion that enough simplifications should be made in order to include all probable effects, instead of overparameterizing particular factors.

One simplification that has been rarely discussed is the assumption of linear correlation between the inhibitory effects and hydrolysis degree [23, 24]. This is obviously correct with product inhibition by sugars, but in the case of changing cellulose hydrolysability or lignin accessibility, the linearity assumption is arbitrary, and the relation may as well be exponential or irregular. In fact, combining linear reversible and quadratic irreversible product inhibition led to almost equal fit (*R*^2^ of 0.9984) compared to the combination of the three inhibitory factors, but with one model parameter less.

While this study is focused on the comparison of the mathematical form of the different models, a larger dataset would be required for accurate determination of the parameters. The iterative fitting of each model was repeated with several combinations of initial values. The standard deviations from mean were calculated for each parameter for the fitting results reaching at least 99 % of the optimum fit, in order to describe how explicitly each parameter was determined (see Additional file 1: Table S1). Particularly large deviations (up to 830 %) were observed with the adsorption parameters *K* and *e*_*m*_, indicating implicit determination and suggesting that the inclusion of Langmuirian adsorption kinetics may not be necessary at the current level of robustness. Accordingly, previous reports of simplified models have also shown good fits with classic Michaelis–Menten-type models [23, 37], in which the proportion of occupied binding sites on the substrate is omitted. A more reliable determination was obtained for the catalytic constants (*k*_cat_) and the inhibitory constants (*α* and *λ*) with the standard deviation ranging from 1 to 110 % for the best fitting models. The fitted catalytic constants were similar for AH- and NaOH-straw in most models, ranging from 20 to 420 mg FPU^−1^ h^−1^ (Additional file 1: Table S1). This suggests that after taking inhibitory mechanisms into account, the hydrolysability of cellulose itself was essentially similar in both materials and the higher hydrolysis rate of NaOH-straw is partly explained by a larger cellulose concentration in the reaction.

## Conclusions

The hydrolysis rates and material properties of autohydrolysed and delignified straw were studied as a function of hydrolysis degree. The hydrolysis rate depended solely on the hydrolysis degree, and there was no difference in the outcome between stepwise or instantaneous enzyme additions. The patterns of residual activity and hydrolysis rates suggested that no particular enzyme denaturation occurred and there were no time-dependent changes in the substrate affecting hydrolysability, other than hydrolysis itself. Some phenolic substances were dissolved during hydrolysis, but they did not contribute to product inhibition or substrate hydrolysability. While product inhibition and reduction of cellulose hydrolysability are known to occur, the dependence of the hydrolysis maximum on the enzyme dosage could only be explained by partial permanent activity loss, suggesting hydrolysis-dependent irreversible inhibition, possibly by non-productive binding. However, hydrolysis did not increase lignin surface area and increase in lignin accessibility was not apparent from the changes in porosity. Therefore other non-productive binding sites such as irregular cellulase structures at cellulose-lignin interfaces may also contribute to irreversible activity loss, which is a fascinating subject for further research.

Cellulose surface area was found to correlate with the total cellulose content and it is thus a suitable approximation for substrate concentration in kinetic modelling of cellulose hydrolysis. A kinetic model of cellulose hydrolysis should account for product inhibition, reduction of hydrolysability and partial permanent activity loss, and the possibility that the latter two may not correlate linearly with hydrolysis. More generally, the level of detail should be balanced between the different factors, instead of extensively describing a particular factor.

## Methods

### Materials

Wheat straw (39.0 % cellulose, 23.7 % xylan, 23.7 % lignin) was autohydrolysed at 180–190 °C, for 20 min, steam exploded and washed as described previously [43]. The material was then ground using a Fritz Pulverisette rotor mill (Fristch, Germany) to pass a 1-mm screen, in order to enable homogenous sampling of the hydrolysis suspension. Whatman 1 filter paper (Sigma–Aldrich) was milled similarly. NaOH-delignified straw described previously [43] was used as such.

### Hydrolysis and sampling

The hydrolysis reactions were performed in duplicates in 1 L Erlenmeyer flasks at 50 °C, pH 5 (50 mM Na-phosphate buffer), 200 rpm at a solid concentration of 5 % in an initial reaction volume of 200 mL. A mixture of enzymes [43] consisting by volume of 85 % cellulase (Econase CE, Roal Oy), 10 % cellobiase (Novozyme 188) and 5 % xylanase (GC 140, Genencor) was used. The mixture contained the activity of 51.0 FPU mL^−1^ according to the Econase CE content, and the protein concentration of 42.9 mg mL^−1^. Tetracycline (4 mg mL^−1^) and cycloheximide (3 mg mL^−1^) were added to prevent microbial contamination. For dye adsorption analysis, sets of 1 mL suspension samples were taken at each time point into glass tubes on ice, centrifuged and washed with 10 mL of degassed, ice-cold Milli-Q water. The supernatant was discarded, and the pellet was weighed and frozen immediately. The water remaining in the pellet was determined from the weight of the pellet, while dry matter was analysed by lyophilisation of a parallel sample. For thermoporometry, 1 mL slurry samples were taken on ice and the solids were separated by centrifugation. The supernatant was recovered for the analysis of sugars, phenols and residual activity. In order to remove dissolved sugars and prevent further hydrolysis, the sample pellet was washed twice with 1.4 mL degassed, ice-cold water, sampled into precooled 50 μl aluminium pans and frozen immediately. All solid samples were stored frozen until analysis and analysed as such in the original wet state.

### Residual cellulase activity and determination of inhibition

The residual cellulase activity in the duplicate hydrolysate supernatant samples was determined by hydrolysis of milled Whatman 1 filter paper at 2 % solids concentration (w/w) in a total volume of 2 mL in 15 mL polypropylene tubes at 50 °C, shaken at 200 rpm in a tilted position for 20 h. A 200-μl hydrolysate sample was applied for the reaction. The reaction was stopped by the addition of 100 μl of 2 M H_2_SO_4_. Then, 100 μl of 25 g L^−1^ mannitol was added as an internal standard, the suspension was mixed thoroughly, centrifuged and the supernatant was analysed for sugars by HPLC without further dilution. The hydrolysis yield was determined from the difference of the known amount of hydrolysate sugars and the final sugar concentration. The hydrolysis yield was converted to a corresponding residual enzyme amount (residual FPU g^−1^ DM in the original hydrolysis reaction) using a non-linear hydrolysis standard (see Additional file 1: Fig. S1) produced in duplicate with fresh enzyme dilutions corresponding to enzyme dosages from 1 to 10 FPU g^−1^ DM in the original hydrolysis reactions.

Similarly, the determination of inhibitory effects of the hydrolysates was performed by the hydrolysis of Whatman 1 at 5 % solid concentration at a total volume of 5 mL in the presence of 3.5 mL of the final hydrolysate supernatant or reference sugar solution with an addition of fresh enzymes (10 FPU g^−1^ Whatman) and a hydrolysis for 6 h. The initial glucose and xylose concentrations were equalised to those of the reaction with the NaOH-straw hydrolysate by an appropriate addition of pure sugars. H_2_SO_4_ and mannitol were added after the reaction in the same proportions as above. The hydrolysis yield was determined from the difference of initial and final sugar concentrations. The experiment was performed twice.

### Analysis of the surface areas of cellulose and lignin

The surface areas of cellulose and lignin on the solid hydrolysis residues were analysed by determining the monolayer adsorption maximum of Congo Red (Direct Red 28) [25] and Azure B [39], respectively. For dye adsorption, 4 mL of dye (Congo Red in 30 mM phosphate buffer, pH 6 and Azure B in 50 mM Na-phosphate buffer, pH 7) was added to the glass tube with the sample pellet and incubated for 24 h on a shaker (200 rpm) in tilted position. In order to minimise further hydrolysis of the samples during incubation, Congo Red adsorption was performed at 65 °C and Azure B adsorption at 15 °C, while previously 60 and 25 °C have been used, respectively. After incubation, the tubes were centrifuged, the supernatant was filtered through a 0.45-µm PTFE filter and the dye concentration was determined spectrophotometrically (Congo Red at 498 nm and Azure B at 647 nm). The adsorbed dye amount was calculated from the residual soluble concentration, corrected with the amount of water in the sample pellet.

An adsorption isotherm was determined before and after each reaction, (and additionally at 1 and 72 h for AH-straw at 10 and 2 FPU, respectively) using Congo Red concentrations of 2, 1, 0.5, 0.15, 0.05 and 0 g L^−1^ and Azure B concentrations of 1, 0.6, 0.3, 0.2, 0.1 and 0 g L^−1^. For optimal fit, Congo Red adsorption was fitted to the BET isotherm as described in our previous work [42] and Azure B adsorption was fitted to the Langmuir isotherm (for isotherms, see Additional file 1: Fig. S2). After determining the isotherm parameters at the beginning and at the end of the reactions, the surface areas in the intermittent time points were determined from the adsorption at a single concentration (1 g L^−1^ Congo Red and 0.6 g L^−1^ Azure B), by assuming that the change in the parameters correlated linearly with hydrolysis.

### Analysis of sugars and phenols

Sugar analysis was performed by HPLC as described previously [43]. Samples were prepared from 100 µl of the hydrolysis supernatants. The HPLC system comprised a Micro-Guard De-Ash pre-column (Bio-Rad, USA), an SPO810 column (Shodex) coupled to a refractive index detector (Shimadzu). Deionised water delivered at a flow rate of 0.7 mL min^−1^ was used to elute the columns at 60 °C.

Phenolic analysis was based on the Folin–Ciocalteau reaction following the micro-scale protocol by Waterhouse [44]. In short, a hydrolysate sample of 20 μl was reacted with commercial Folin–Ciocalteau reagent (Merck) and NaCO_3_ in a plastic cuvette in a total volume of 2 mL. The phenol concentration was determined spectrophotometrically at 765 nm against a gallic acid standard as mg mL^−1^ gallic acid equivalent (GAE).

### DSC thermoporometry

The pore size distribution (wet porosity) of the hydrolysed solids was analysed by DSC thermoporometry using PerkinElmer DSC6000. The method is based on the melting-point depression of water confined in pores, which allows the stepwise analysis of the volumes of pores of different diameters by calorimetrically determining the volume of melting water at sub-zero temperatures. The method was adapted from Park et al, [45], applying the corrections for the sample heat capacity according to Driemeier et al, [46]. A 13-step temperature programme from −35 to 10 °C was applied as described in detail in our recent work [42]. After DSC analysis, the pans were perforated and lyophilised for sample dry matter determination.

### Kinetic modelling

Modelling was performed using Matlab R2010b (Mathworks). The kinetic models were fitted to the hydrolysis time curves [24, 32] by non-linear regression (*lsqcurvefit*), where the time curve was numerically integrated from the kinetic model as a nested function (*ode15s*). The hydrolysis rate curves (Fig. 1b) were obtained by individually fitting the hydrolysis model with product inhibition (Eqs. 3–6) to each hydrolysis curve (overall *R*^2^ = 0.99983). For kinetic studies, all data were fitted simultaneously, enabling constant parameters across reactions. In order to verify global optimum in parameter iteration, fitting was repeated with a three-level full factorial set of initial parameter values (initial values 0.01, 1 and 100). Thus, 3^n^ repetitions were performed for fitting n parameters. The optimum fit is reported and for each model, the parameters of the optimum fit and the standard deviation of the parameter values for the repetitions reaching at least 99 % of the optimum fit are presented in the Additional file 1: Table S1.

## Additional file

10.1186/s13068-016-0431-3 Modelling details. Fitted optimum parameters and the parameter standard deviations of the best fitting parameter sets. **Figure S1.** Non-linear hydrolysis standard. **Figure S2.** BET-isotherms of Congo Red adsorption and Langmuir-isotherms of Azure B adsorption.

## Abbreviations

AH-straw: autohydrolysed straw
NaOH-straw: NaOH-delignified straw
DM: dry matter
GAE: gallic acid equivalent
FPU: filter paper unit
