# Supplementary material for: Rate-constraining changes in surface properties, porosity and hydrolysis kinetics of lignocellulose in the course of enzymatic saccharification
Source: Biotechnol Biofuels. 2016 Jan 26;9:18. doi: 10.1186/s13068-016-0431-3 (PMC4727270; doi:10.1186/s13068-016-0431-3)
Supplement: Supplementary file 1 — 10.1186/s13068-016-0431-3 Modelling details. Fitted optimum parameters and the parameter standard deviations of the best fitting parameter sets. Figure S1. Non-linear hydrolysis standard. Figure S2. BET-isotherms of Congo Red adsorption and Langmuir-isotherms of Azure B adsorption. [file 13068_2016_431_MOESM1_ESM.docx]

**Rate constraining changes in surface properties, porosity and hydrolysis kinetics of lignocellulose in the course of enzymatic saccharification**

Ville Pihlajaniemi*, Mika Henrikki Sipponen, Anne Kallioinen, Antti Nyyssölä, Simo Laakso

Aalto University, School of Chemical Technology, P.O. Box 16100, FI-00076 Espoo.

*E-mail: ville.pihlajaniemi@aalto.fi.

**Supplementary information**

Contents

[Model parameters 2](#_Toc436656683)

[Non-linear hydrolysis standard 3](#_Toc436656684)

[Dye adsorption isotherms 4](#_Toc436656685)

[Modelling details: Deriving equation 4. 5](#_Toc436656686)

# Model parameters

The parameters of the optimum fit for each model are listed in Table S1. The iterative fitting of each model was repeated with several combinations of different initial values and the optimum fit is reported. For the best parameter sets (at least 99% of the optimum fit), the standard deviation from mean was calculated for each parameter.

**Table S1.** Modelling details. Fitted optimum parameters and the parameter standard deviations of the best fitting parameter sets.

| **Model (Equations applied)** | **R^2^** | **Repetitions**  **(best fits)** | **Parameters (standard deviations)** | | | | | | | |
| --- | --- | --- | --- | --- | --- | --- | --- | --- | --- | --- |
|  |  |  | K  mL/FPU | e_m_  FPU/g | k_cat,AH_  mg/(FPU*h) | k_cat,NaOH_  mg/(FPU*h) | α_Rev_  1 | α_Hydrlty_  mg/(FPU*h) | α_Irrev_  FPU/ml | λ  1/h |
| No inhibition (3,4) | 0.8431 | 81 | 0.023818 | 0.026539 | 102.17 | 97.369 |  |  |  |  |
|  |  | *(32)* | *(134%)* | *(322%)* | *(50%)* | *(55%)* |  |  |  |  |
| Reversible product inhibition (3,4,5,6) | 0.9753 | 243 | 0.17650 | 0.020767 | 393.87 | 421.24 | 66.400 |  |  |  |
|  |  | *(67)* | *(130%)* | *(360%)* | *(60%)* | *(60%)* | *(68%)* |  |  |  |
| Reducn. Of hydrolysability (3,4,5,9) | 0.9584 | 243 | 0.0044135 | 0.31673 | 111.29 | 112.33 |  | 148.08 |  |  |
|  |  | *(130)* | *(103%)* | *(272%)* | *(82%)* | *(82%)* |  | *(83%)* |  |  |
| Irreversible product inhibition (3,4,5,7) | 0.9549 | 243 | 0.019568 | 0.056636 | 268.28 | 142.40 |  |  | 0.68135 |  |
|  |  | *(71)* | *(147%)* | *(478%)* | *(135%)* | *(129%)* |  |  | *(1%)* |  |
| Dentaturation (3,4,10) | 0.9747 | 243 | 1.2243 | 0.0006171 | 389.24 | 319.08 |  |  |  | 0.084213 |
|  |  | *(93)* | *(73%)* | *(512%)* | *(115%)* | *(117%)* |  |  |  | *(12%)* |
| Time dependent irreversible product inhibition (3,4,5,7,8) | 0.9826 | 729 | 0.047642 | 0.035456 | 139.89 | 89.755 |  |  | 0.81998 | 0.84736 |
|  |  | *(17)* | *(148%)* | *(398%)* | *(66%)* | *(67%)* |  |  | *(7%)* | *(63%)* |
| Reversible product inhibition & denaturation (3,4,5,6,10) | 0.9967 | 729 | 129.38 | 0.029883 | 51.511 | 68.624 | 77.130 |  |  | 0.035783 |
|  |  | *(291)* | *(878%)* | *(269%)* | *(110%)* | *(89%)* | *(100%)* |  |  | *(17%)* |
| Irreversible product inhibition and reduction of hydrolysability (3,4,5,7,9) | 0.9896 | 729 | 0.19609 | 0.050139 | 33.528 | 30.086 |  | 41.645 | 0.30045 |  |
|  |  | *(317)* | *(330%)* | *(202%)* | *(110%)* | *(107%)* |  | *(106%)* | *(5%)* |  |
| Reversible product inhibition and squared irreversible product inhibition (3,4,5,5^squared^,7) | 0.9984 | 729 | 5.7198 | 15.010 | 52.657 | 55.540 | 68.282 |  | 0.88337 |  |
|  |  | *(247)* | *(216%)* | *(325%)* | *(53%)* | *(54%)* | *(60%)* |  | *(2%)* |  |
| Reversible and irreversible product inhibition and reduction of hydrolysability (3,4,5,6,7,9) | 0.9990 | 2187 | 5512.6 | 0.025019 | 19.526 | 19.028 | 22.232 | 11.437 | 0.28801 |  |
|  |  | *(699)* | *(827%)* | *(263%)* | *(87%)* | *(77%)* | *(78%)* | *(105%)* | *(6%)* |  |

# Non-linear hydrolysis standard

**Figure S1.** Non-linear hydrolysis standard.

# Dye adsorption isotherms

The Brunauer Emmett Teller (BET) isotherm, based on the Langmuir theory, but extended to cover multilayer adsorption, was applied for fitting the adsorption isotherms of Congo Red in accordance with the tendency of Congo Red to aggregate (R^2^ = 0.993). Langmuir isotherm applied for the adsorption of Azure B (R^2^ = 0.979).


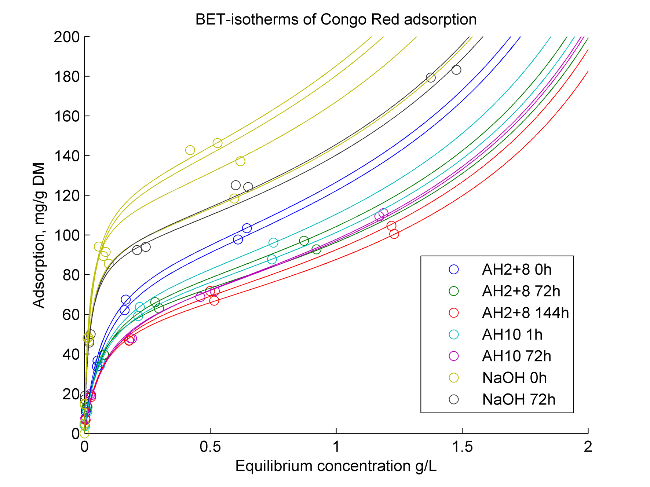

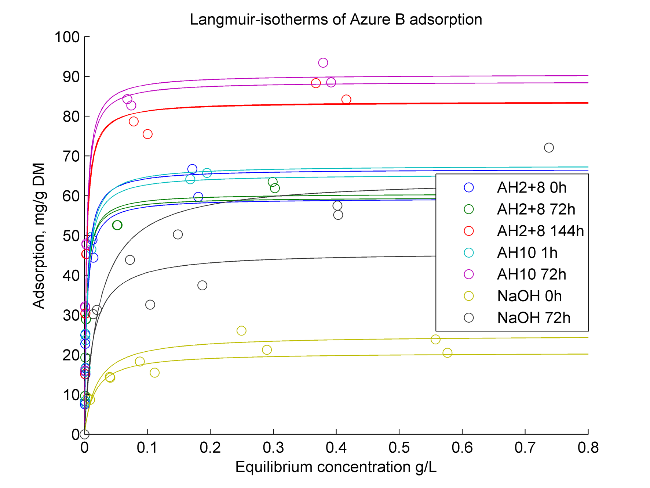


**Figure S2.** BET-isotherms of Congo Red adsorption and Langmuir-isotherms of Azure B adsorption.

# Modelling details: Deriving equation 4.

The solution for the concentration of enzyme-substrate complexes $[ES]$ (Eq. 4) is derived by substituting ${[E}_{F}]={[E}_{o}]-\left[ ES \right]$ to the equation 2, which leads to equation S1.

$$\left[ ES \right]=\frac{\left[ S \right]e_{m}K\left( \left[ E_{o} \right]-\left[ ES \right] \right)}{1+K\left( \left[ E_{o} \right]-\left[ ES \right] \right)} (S1)$$

The equation S1 can be arranged to obtain the quadratic equation S2.

$$-K\left[ ES \right]^{2}+\left( 1+K\left[ E_{o} \right]+\left[ S \right]e_{m}K \right)\left[ ES \right]-\left[ S \right]e_{m}K\left[ E_{o} \right]=0 (S2)$$

Solving [ES] from the equation S2 by using the quadratic formula leads to potentially 2 solutions (Eq. S3)

$$[ES]=\frac{-b\pm\sqrt{b^{2}-4ac}}{2a} (S3)$$

$$a=-K; b=1+K{[E}_{0}]+[S]e_{m}K; c=-[S]e_{m}K{[E}_{0}].$$

First we will define that $\left\{ K,E_{o},e_{m},S>0 \right\}$ and denote $\alpha=\left[ S \right]e_{m}$ and ${E=[E}_{o}]$ for clarity. The discriminant $\Delta$ of the equation S3 can be rearranged into a quadratic function (Eq. S4), where the linear coefficient and the constant are positive and the quadratic coefficient is equal or larger than zero. Thus, the discriminant is larger than 1 and equation S3 does have two solutions.

$$\Delta=K^{2}\left( E-\alpha\right)^{2}+K\left( 2E+2\alpha\right)+1>1 (S4)$$

The correct solution must be chosen for modelling. First we realize that $\left[ ES \right]$ cannot be smaller than zero or larger than total enzyme amount $E$ or the total amount of binding sites $\alpha$ (Eq. S5).

$$0\leq\left[ ES \right]=\frac{-b\pm\sqrt{\Delta}}{-2K}\leq\left\{ E,\alpha\right\} (S5)$$

First, we inspect the statement that the first solution is higher than zero (Eq. S6). This inequality can be arranged into positive terms (Eq. S7), which allows raising both sides to the second power. Observing the result (Eq. S8), we find that the statement is correct.

$$\frac{-b+\sqrt{\Delta}}{-2K}=\frac{1+K\left( E+\alpha\right)}{2K}-\frac{\sqrt{\Delta}}{2K}>0 (S6)$$

$$1+K\left( E+\alpha\right)>\sqrt{\Delta} (S7)$$

$$K^{2}\left( E+\alpha\right)^{2}+K\left( 2E+2\alpha\right)+1>K^{2}\left( E-\alpha\right)^{2}+K\left( 2E+2\alpha\right)+1 ∎ (S8)$$

Next, we confirm that the first solution cannot be higher than $E$ or $\alpha$. The solution can be arranged to the form of S9, where all terms are positive. By discarding the K-containing terms from the denominator, we arrive at an inequality stating that the largest obtainable value is the smaller from $E$ and $\alpha$. Thus the solution is always within the defined possible range.

$$\frac{-b+\sqrt{\Delta}}{-2K}=\frac{1}{2}\left( \frac{1}{K}+E+ \alpha-\sqrt{\left( E-\alpha\right)^{2}+\frac{2\left( E+\alpha\right)}{K}+\frac{1}{K^{2}}} \right)$$

$$=\frac{\left( \frac{1}{K}+E+ \alpha\right)^{2}-\left( E-\alpha\right)^{2}-\frac{2\left( E+\alpha\right)}{K}-\frac{1}{K^{2}}}{2\left( \frac{1}{K}+E+ \alpha+\sqrt{\left( E-\alpha\right)^{2}+\frac{2\left( E+\alpha\right)}{K}+\frac{1}{K^{2}}} \right)}$$

$$=\frac{2E\alpha}{\frac{1}{K}+E+ \alpha+\sqrt{\left( E-\alpha\right)^{2}+\frac{2\left( E+\alpha\right)}{K}+\frac{1}{K^{2}}}}\leq\frac{2E\alpha}{E+\alpha+\left| E-\alpha\right|} \left( S9 \right)$$

$$\frac{2E\alpha}{E+\alpha+\left| E-\alpha\right|}=\frac{2E\alpha}{2\max\left\{ E,\alpha\right\}}=\min\left\{ E,\alpha\right\} ∎ (S10)$$

Finally, we study the second solution. The second solution only includes positive terms (Eq. S11) and we observe that the square root term is larger than the absolute value of the difference of $E$ and $\alpha(Eq. S12)$. Thus we find that the solution is larger than $E$ and $\alpha$, which is impossible (Eq. S13).

$$\frac{-b-\sqrt{\Delta}}{-2K}=\frac{1}{2}\left( \frac{1}{K}+E+ \alpha+\sqrt{\left( E-\alpha\right)^{2}+\frac{2\left( E+\alpha\right)}{K}+\frac{1}{K^{2}}} \right) (S11)$$

$$\sqrt{\left( E-\alpha\right)^{2}+\frac{2\left( E+\alpha\right)}{K}+\frac{1}{K^{2}}}>\left| E-\alpha\right| (S12)$$

$$\frac{1}{2}\left( \frac{1}{K}+E+\alpha+\left| E-\alpha\right| \right)>\left\{ E,\alpha\right\} ∎ (S13)$$
